# Trace metals contamination potential and health risk assessment of commonly consumed fish of Perak River, Malaysia

**DOI:** 10.1371/journal.pone.0241320

**Published:** 2020-10-26

**Authors:** Mohammed Abdus Salam, Shujit Chandra Paul, Rabiatul Adawiyah M. Mohamad Zain, Snahasish Bhowmik, Mithun Rani Nath, Sadia Afrin Siddiqua, Tutun Das Aka, Muhammad Anwar Iqbal, Wan Rashidah Kadir, Rozita Binti Ahamad, Md. Abdul Khaleque, Aweng Eh Rak, Mohamad Faiz Mohd Amin

**Affiliations:** 1 Department of Environmental Science and Disaster Management, Noakhali Science and Technology University, Noakhali, Bangladesh; 2 Faculty of Earth Science, University Malaysia Kelantan, Jeli Campus, Jeli, Kelantan, Malaysia; 3 Department of Applied Chemistry and Chemical Engineering, Noakhali Science and Technology University, Noakhali, Bangladesh; 4 Department of Animal Breeding and Genetics, Bangladesh Agricultural University, Mymensingh, Bangladesh; 5 Department of Pharmacy, Noakhali Science and Technology University, Noakhali, Bangladesh; 6 Forest Biotechnology Division, Forest Research Institute Malaysia (FRIM), Kepong, Selangor, Malaysia; 7 School of Environmental Science and Management, Independent University, Bangladesh, Dhaka, Bangladesh; Chinese Academy of Sciences, CHINA

## Abstract

The rapid growth of industrial and agricultural activities in Malaysia are leading to the impairment of most of the rivers in recent years through realising various trace metals. This leads to toxicity, particularly when the toxic has entered the food chain. Perak River is one of the most dynamic rivers for the Malaysian population. Therefore, in consideration of the safety issue, this study was conducted to assess the concentration of such metals (Cd, Cu, Zn, Fe, and Pb) in the muscles of most widely consumed fish species (*Barbonymus schwanenfeldii*, *Puntius bulu*m, *Puntius daruphani*, *Hexanematichthys sagor*, *Channa striatus*, *Mystacoleucus marginatus*, and *Devario regina*) from different locations of Perak River, Malaysia by employing inductively coupled plasma optical emission spectroscopy (ICP-OES). Among the trace metals, Fe and Cd were found to be the highest (29.33–148.01 μg/g) and lowest (0.16–0.49 μg/g) concentration in all of the studied species, respectively. Although the estimated daily intakes (μg/kg/day) of Cd (0.65–0.85), Fe (79.27–352.00) and Pb (0.95–12.17) were higher than their reference, the total target hazard quotients values suggested that the local residents would not experience any adverse health effects from its consumption. In contrast, the target cancer risk value suggested that all fish species posed a potential cancer risk due to Cd and cumulative cancer risk values, strongly implying that continuous consumption of studied fish species would cause cancer development to its consumers.

## Introduction

Trace metal, originating from various natural and anthropogenic activities around the world, is now considered as one of the main pollutants for aquatic environments [1, 2]. Although some essential trace metals like Fe, Cu, Zn play important roles in body functions, most of the nonessential trace metals, such as Cr, Pb, Hg, and Cd, are usually considered as toxic due to their harmful effects after entering human biological systems [3]. These trace metals usually accumulate in the human body through the consumption of aquatic animals, mostly fish and shellfish, which act as an intermediate sink to such trace metals [4].

However, fish is one of the major sources of protein in the world, containing essential fat, vitamins, and minerals [5]. The world per capita fish supplies have increased from 18.1 kg in 2009 to about 20.3 kg in 2016 [5, 6]. On the other hand, the intake of fish in Malaysia has surprisingly increased by at least 150% from 1961 to date. The average Malaysian now consumes approximately 57 kg of seafood per year [7, 8].

The trace metals were generated from various sources gathered in the water body and sediment, though sediment acts as a superior basin for them compared to the water itself [9]. However, trace metals tend to accumulate in aquatic animals such as fish with long-term exposure in the marine environment. Furthermore, such bioaccumulation of trace metals in fish also depends on the pH, temperature, salinity of the water body as well as age, size, sex, physiological conditions, feeding types, habitat types, trophic level, route of uptake and physiological function of organs of aquatic animals [10–12]. The toxicity of these trace metals has proven to be a major threat, and there are numerous health impacts associated with it. The consumption of excess trace metal contained fish can malfunction the human digestive, cardiovascular and central nervous system [13]. In particular, prolonged exposure to metals such as Cd, Pb, and Cr, can cause cancer, liver damage, and eventual death of living organisms [14] Therefore, it is essential to study the trace metal concentration in fish species along with their both cancerous and noncancerous health risks to assure their harmless consumption.

Malaysia is a developing country dealing with rapid urbanisation, population growth, and industrialisation in the last few years [15–17]. Such types of growth activities, along with deforestation, domestic or animal farming sewage, sand mining, and agricultural activities act as the main sources of trace metal pollution of Malaysia's aquatic environment [18, 19]. The Perak River, which is the source of drinking water, and edible aquatic species are also being polluted by trace metals from these activities [20]. Such pollution leads to the aggregation of trace metals in aquatic species, and sooner or later it will affect their consumers worldwide, predominantly to the local people. Furthermore, the country is a popular place for tourists worldwide and, in every year, it exports fish abroad. Therefore, considering the food safety of Malaysians as well as the world population, this study was designed to analyse the bioaccumulation of trace metals in the widely edible portions (flesh or muscle) of commonly consumed fish species and to conduct health risk assessment caused by their consumption.

Although several studies, regarding trace metal accumulation in fish species and their risk assessment, were done all around Malaysia, no such study was performed to assess the risk of commonly consumed fish of Perak river [21–23]. As Perak river is engaged with various industrial, agricultural and residential activities, therefore, there is a strong probability of accumulation of trace metals in the river's aquatic organisms. Thus, the primary objectives of this study are to assess the accumulation characteristics of trace metals in seven types of commonly consumed freshwater fish species; namely, tinfoil barb (*Barbonymus schwanenfeldii*), crossbanded barb (*Puntius bulu*), lemon fin barb (*Puntius daruphani*), sagor catfish (*Hexanematichthys sagor*), striated snakehead (*Channa striatus*), barb spiny, (*Mystacoleucus marginatus*), and fowler's danio (*Devario regina*) of the Perak River and to elucidate the possible harmful effects of trace metals through their consumption.

## Materials and methods

### Study area

**Fig 1:**
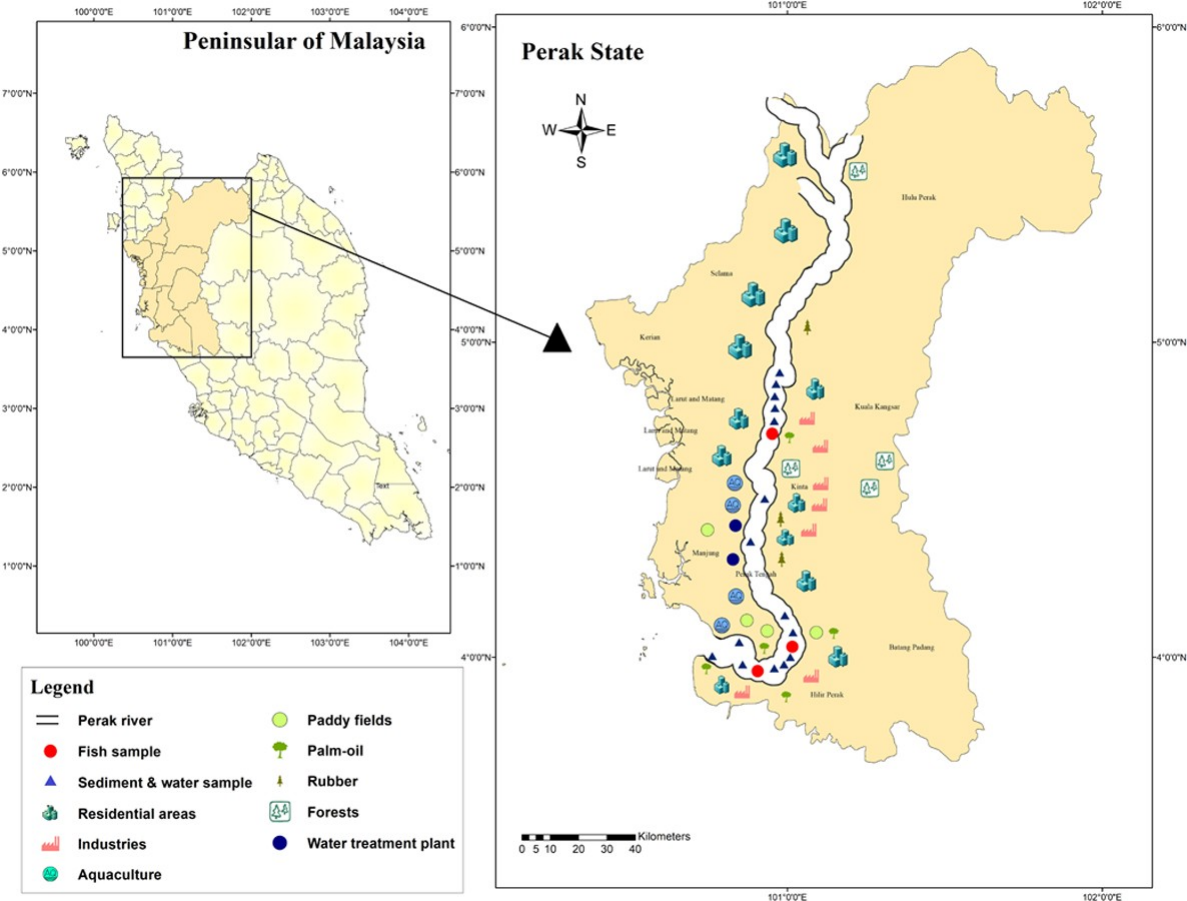
The map of Perak River. The samples were collected along the river according to the availability of the fish from the upstream to downstream (Source: USGS National Map Viewer [28]).

Sungai Perak, the second-longest river in Peninsular Malaysia is known as the ‘River of Life' for Perak, Malaysia as the river acts as a water source for the state people and also use for recreational activities, fishery, mining, irrigation of paddy fields, agricultural farming and industrial activities surrounding it (Fig 1) [24]. The river covers approximately 400 km in a 15,000 km^2^ catchment, which is almost 70% of the total land area of Perak state. As the river starts from the north-western corner of the state, Perak River flows south to Teluk Intan, where it bends westward and then flows into the Channels of Malacca [24]. The source of Perak River is the mountainous Perak-Kelantan-Thailand border of the Belum Forest Reserve. Some of the branches of the river include the Bidor River and the Kinta River [25]. Geological studies have shown that the Perak river has been developing with time, and it shifted to the southern direction resulting in partial silting in the estuary [26]. The coastal areas of Perak are usually covered by Quaternary sediments having different lithologies like peat, clay, silt, sand and gravel [27]. Geomorphologically, this area is a low lying area with a range of 0–10 metres from the mean sea level [26].

### Ethical statement

Only the fresh- and good-looking fish were collected from the local fishermen. None of the sampled species was endangered or protected by the Govt. No permit was required to conduct the present study. There were no ethical considerations linked to the experiment.

### Sample collection and preparation for trace metals analysis

A total of 110 fish samples were collected from the Perak River during the dry season in August 2015 from three different locations of Perak river, as shown in Fig 1. The map was drawn by using ArcGIS software (version 10.1). There were seven species of fish collected in the present study: tinfoil barb (*Barbonymus schwanenfeldii*), crossbanded barb (*Puntius bulu*), lemon fin barb (*Puntius daruphani*), sagor catfish (*Hexanematichthys sagor*), striated snakehead (*Channa striatus*), barb, spiny (*Mystacoleucus marginatus*), and fowler's danio (*Devario regina*). Only the fresh- and good-looking fish were separated from the stock and marked for future experiments. The length and weight of the fish samples were measured by a fish ruler and recorded once they had arrived at the laboratory. Fish samples were washed using running tap water and thawed at normal temperature prior to the analysis. All of the fish species were dissected on a clean bench shortly with a stainless-steel knife which had been sterilised with acetone and hot distilled water. Only the flesh muscles tissue of the fish was removed, placed in glass bottles, and frozen for metal analysis. The samples were then freeze-dried at -20°C for 24 h, and each sample was blended homogenously until the sample turned into powdered form and then packed and sealed in polythene bags separately before undergoing acid digestion processes. After that, 0.5 g samples were weighed and put into a 50 ml beaker. 6 ml of (65%) concentrated nitric acid (HNO_3_) was added into the beaker, followed by adding 2ml of (30%) hydrogen peroxide (H_2_O_2_). All the beakers were placed on the hot plate separately at 60˚C for 2 hours until they became dry to ensure complete digestion of all organic matters. Three per cent (3%) of diluted nitric acid (HNO_3_) was dropped into the beakers, which were placed on the hot plate after 20 minutes [29]. The digested solutions were left to cool down in ambient temperature. After cooling, the digested solutions were filtered through 0.45 μm Whatman filter paper into falcon tubes and double rinsed with deionised water to ensure that the entire digest was transferred into the tube. The filtrated samples were augmented to 30 ml by adding Mili-Q deionised water for dilution. The determination of Cu, Cd, Pb, Fe, and Zn in fish muscle tissue was conducted using inductively-coupled plasma spectrometry (ICP-OES) (Varian 725-ES, Australia). The concentrations of trace metals were expressed as μg/g wet weight. As a part of quality assurance and quality control procedural blank (additional 6 ml of concentrated nitric acid and 2 ml of hydrogen peroxide only), along with samples and certified reference materials (CRMs), was carried out to obtain accurate result during heavy metals analysis in each species of fish which has led to a meaningful data for the present study. All of the experiments were carried out in three replicates to eliminate any batch errors, and only average values with standard deviation were reported. Matrix Spike was prepared for every batch of digestion samples to increase the confidence in the accuracy and validity of the sample test results. The analysed samples were spiked and ran in ICP-OES, and the concentrations of the metal contents were determined from the calibration curves, which varied from 98.5% to 119%. The Limit of detection (LOD) values of Cd, Cu, Pb, Zn and Fe were 0.002 μg/g, 0.14 μg/g, 0.01 μg/g, 0.21 μg/g and 0.002 μg/g respectively. The calibration of ICP-OES was done by using a multi-element standard solution. All the reagents used in this study were analytical grade and purchased from Merck, Darmstadt, Germany.

### Biota-sediment accumulation factors (BSAFs)

Average metal concentrations in fish species from the present study and Average metal concentrations in sediments from another study which was done simultaneously with the present study [30] were used to calculate the biota-sediment accumulation factors (BSAF). The BSAF is a parameter that designates bioaccumulation of sediment-associated organic compounds or metals into tissues of various ecological receptors [31]. BSAFs were calculated using (Eq 1): [32].

$$BSAF=\frac{C_{b}}{C_{s}}$$ (1)

Where: C_b_ is the concentration of a contaminant in biota (fish) samples (μg/g), while C_s_ is the concentration of the contaminant in the sediment (μg/g), which was determined in our previous study [33]. Biota-sediment accumulation factors (BSAFs) help to assimilate the bioaccumulation rates among the tissues of the fish species [32].

### Bioaccumulation factor (BAF)

Bioaccumulation factor (BAF) is the equivalence ratio between the metal concentration in any organ (tissues) of a biota (fish) and metal concentration in the ambient media (water) [34]. Bioaccumulation factor (BAF) acts as a pollution-scale independent parameter (a function of basic environmental variables), as it can be calculated from data measured in field studies. It was calculated using (Eq 2): [35]

$$BAF=\frac{{\left[X\right]}_{organism}}{{\left[X\right]}_{water}}$$ (2)

where BAF is bioaccumulation factor, L/kg, *[X]organism* is the concentration of the element X (trace metal) in the examined organism (fish), mg/kg; *[X]water* is the concentration of X (trace metal) in the water environment, mg/L as determined in our previous study during August of 2015 [30].

### Health risk assessment

#### Estimated daily intake of trace metals

The estimated daily intake (EDI) of trace metals by humans is evaluated according to the mean metal concentration in fish and the amount of daily fish consumption. The EDIs of metals were determined by Eq 3:

$$EDI=\frac{metal\;concentration\;\left(\mu g/g\;.\right)\;x\;consumption\;rate\;\left(g/d\right)}{body\;weight\;\left(kg\right)}$$ (3)

where bodyweight for adults is considered to be 64 kg concerning to Malaysian and was derived from numerous local Malaysia studies [25]. The consumption rate was 160 g/d/person for Malaysian adults [36, 37]. The oral reference dose (RfD) was used to evaluate the EDIs of metals in the fish [38].

#### Target hazard quotients

Current non-cancer risk assessment methods are typically based on the employment of the target hazard quotient (THQ), a ratio between the estimated dose of a contaminant and the reference dose below whereby there will be no apparent risk. The THQ was determined using Eq 4 [38]:

$$THQ=\frac{EF\;x\;ED\;x\;FIR\;x\;C}{RfD\;x\;WAB\;x\;TA}\;x\;10^{-3}$$ (4)

Where EF is the exposure frequency (365 d/year); ED is the exposure duration (70 years), equivalent to the average lifetime; FIR is the food ingestion rate (160 g/d/person for Malaysian adults); C is the metal concentration in the muscle of fish (μg/g wet weight); WAB is the average bodyweight that is 64 kg for Malaysia and was derived from numerous local Malaysian studies [29], and TA is the average exposure time for noncarcinogens (365 d/year x ED) [11]. RfD is the reference dose of the metal (0.5 μg/kg/day for Cd; 4.0 μg/kg/day for Pb; 40 μg/kg/day for Cu; 70 μg/kg/day for Fe; and 300 μg/kg/day for Zn) [34]. A THQ value higher than 1 is usually considered as harmful for consumers [39].

#### Total target hazard quotients

The total target hazard quotient (TTHQ) value was considered to have evaluated whether more than one metal could cause any noncarcinogenic effect to the consumer or not. It was calculated using Eq 5 [40]:

$$TTHQ=THQ_{Cd}+\;THQ_{Cu}+\;THQ_{Fe}+\;THQ_{Zn}+\;THQ_{Pb}$$ (5)

#### Target cancer risk

Cancer risk is utilised to determine the probability of a particular carcinogen causing cancer over a lifetime exposure. Target risk was calculated using Eq 6:

$$TR=\frac{EF\;x\;ED\;x\;FIR\;x\;C\;x\;CSF}{WAB\;x\;TA}\;x\;10^{-3}$$ (6)

where the CSF is the oral carcinogenic slope factor. The CSF for Pb was 8.5 x 10^−3^ (μg/g/day)^–1^, and for Cd, it was 6.3 (μg/g/day)^–1^ [41]. The other parameters were defined previously. The cumulative cancer risk is the summation of the individual trace metal risks and calculated through Eq 7 [42]:

$$\sum TR=\;TR_{Pb}+\;TR_{Cd}$$ (7)

### Statistical analysis

Statistical analysis was conducted using Microsoft EXCEL 2013 and SPSS 20. Pearson correlations were performed to evaluate significant relationships between different species of trace metals. Health risk analysis was conducted by comparing the standard values suggested. Fulton condition factor (K) was calculated (Table 1) to determine the physical condition of sample fish according to Eq 8 [43]:

$$K=100\;\left(\frac{W}{L^{3}}\right)$$ (8)

where W is the weight of fish in gram (gm), and L is the length of fish in centimetres (cm).

**Table 1:** List of fish species with length, weight, and Fulton's condition factor (K) along with ecological characteristics.

Local Name	Common Name	Scientific Name	Sample size (n)	Mean Weight (g) (mean ± SD)	Mean length (cm) (mean ± SD)	K (mean ± SD)
Tengalan	Crossbanded barb	*Puntius bulu*	9	430 ± 20	35 ± 5	1.07 ± 0.41
Kerai	Lemon fin barb	*Puntius daruphani*	9	230 ± 30	27 ± 4.4	1.24 ± 0.45
Sia	Barb, spiny	*Mystacoleucus marginatus*	9	45 ± 7.5	16 ± 4	1.31 ± 0.79
Lampam sungai	Tinfoil barb	*Puntius schwanenfeldii*	15	110 ± 13.5	21 ± 5	1.41 ± 0.85
Seluang pipih	Fowler's danio	*Devario regina*	9	6 ± 2.5	12 ± 2.5	0.31 ± 0.04
Duri	Sagor catfish	*Hexanematichthys sagor*	9	55 ± 10	18 ± 3	1 ± 0.32
Haruan	Striated snakehead	*Channa striatus*	18	50 ± 12	18 ± 4.5	0.98 ± 0.51

## Results and discussion

### Physical characteristics of fish species

Table 1 indicates the physical conditions of the sampled fish species along with their ecological characteristics. The physical conditions of the fish species were considered because the trace metal accumulation depends on age, body weight, and feeding habits [44, 45]. A Fulton's factor closes to, or more than one indicates that the fish was in physically good condition for analysis [46].

### Metal concentrations in fish species

It is well known that trace metals can accumulate in fish tissues through different organs [45]^.^ For this study, muscle tissue from seven different types of fish species was chosen, considering that the fish muscle is the major edible part for consumers. The concentration of five trace metals (Cd, Cu, Pb, Fe, and Zn) in the muscles of seven fish species are listed in Table 2.

**Table 2:** Mean (± SD) metal concentration (μg/g) in fish species of Perak River (n = 3).

Fish Species	Metal Concentration (μg/g wet weight)					
		Cd	Cu	Fe	Zn	Pb
*Puntius daruphani*	Average	0.28 ± 0.09	1.43 ± 0.33	52.25 ± 1.96	33.57 ± 4.09	1.75 ± 0.35
	Range	0.18–0.34	1.09–1.75	51.09–54.51	29.18–37.29	1.38–2.08
*Barbonymus schwanenfeldii*	Average	0.29 ± 0.08	1.34 ± 0.22	38.24 ± 8.35	24.60 ± 4.60	3.96 ± 0.08
	Range	0.23–0.39	1.10–1.54	32.43–34.49	19.28–27.11	3.90–4.02
*Puntius bulu*	Average	0.34 ± 0.13	1.56 ± 0.67	31.71 ± 1.74	18.27 ± 1.52	0.38 ± 0.11
	Range	0.27–0.49	0.79–1.94	29.71–32.55	16.68–18.40	0.30–0.45
*Hexanematichthys sagor*	Average	0.26 ± 0.01	0.31± 0.05	32.75 ± 3.00	36.43 ± 3.16	2.42 ± 0.83
	Range	0.25–0.28	0.06–0.34	29.33–34.99	33.50–39.77	1.83–3.01
*Channa striatus*	Average	0.27 ± 0.09	1.92 ± 0.16	140.80±11.36	58.92 ± 2.84	1.43 ± 0.29
	Range	0.17–0.34	1.75–2.05	127.70–148.01	55.67–60.14	0.59–1.64
*Mystacoleucus marginatus*	Average	0.28 ± 0.04	1.18 ± 0.18	88.11± 2.10	31.34 ± 1.06	1.26 ± 0.55
	Range	0.23–0.31	0.97–1.29	86.56–90.50	30.4–32.50	0.87–3.55
*Devario regina*	Average	0.28 ± 0.1	1.15 ± 0.10	92.45±3.14	84.28 ± 9.31	4.87± 1.46
	Range	0.16–0.42	0.69–1.27	52.60–96.07	73.21–98.22	3.13–6.09
Permissible Limit	MFR (1985) [47]	1	30	-	100	2
	FAO/WHO (1984) [48]	0.2	10	300	150	1.5
	China National Standard [49]	0.1	50	-	-	0.5
	England / MAFF (2000) [50]	0.2	20	-	50	2

Among all the metals, Fe (29.328–148.008 μg/g) was found to be the highest concentration in every species except for *Hexanematichthys sagor*, whereas the concentration of Cd (0.162–0.492 μg/g) was at the lowest for all types of fish species and the similar results were also observed for *Oreochromis niloticus* (tilapia fish) from Langat river (Table 3), and unlike *Oreochromis niloticus*, the four studied fish species; namely, *Puntius daruphani*, *Barbonymus schwanenfeldii*, *Mystacoleucus marginatus*, *Puntius bulu*, and *Devario regina* are also omnivorous [29]. Generally, the mean concentrations of trace metals in these fish species were in the following descending order of magnitude: Fe > Zn > Pb > Cu > Cd. For *Channa striatus* and *Puntius bulu* species, the concentration of Cu was higher than Pb, whereas, for *Hexanematichthys sagor* species, the concentration levels of Zn (36.434 ± 3.157 μg/g) was higher than Fe (32.75 ± 3.009 μg/g). *Devario regina* tends to accumulate the highest concentration of Zn (84.285 μg/g), whereas the lowest concentration of Zn (18.268 μg/g) was found in *Puntius bulu* as compared to other species. The concentration levels of Zn determined in all fish samples were far below the permissible limit recommended by the Malaysian Food Regulation and FAO/WHO. However, according to MAFF (2000), the Zn concentration of *Channa striatus* and *Devario regina* has exceeded the permissible limit (50 μg/g) (Table 2).

**Table 3:** Comparison of trace metals concentrations (μg/g) in freshwater fish species collected from different parts of the world.

Area	Species	Cd	Cu	Fe	Zn	Pb	Reference
Langat River and Engineering Lake, Bangi, Malaysia	*Oreochromis niloticus*	0.05–0.03	1.69–1.01	-	26.13–20.58	0.26–0.99	[29]
Pahang River, Basin, Malaysia	*Channa striatus*	-	2.24	-	1.82	0.01	[51]
Chini Lake, Peninsular Malaysia	*Puntius bulu*	0.14	0.28	-	2.73	0.98	[52]
Kelantan River, Malaysia	*Barbonymus schwanenfeldii*	0.03				0.10	[53]
	*Puntioplites bulu*	0.04	-	-	-	0.07	
	*Tachysurus maculatus*	0.05				0.16	
Paira River, Bangladesh	*Channa striata*	0.01–0.03	0.2–2.2	-	-	0.40–1.00	[45]
						0.20–1.10	
	*Channa punctate*	0.01–0.04	0.4–1.6				
Pearl River Delta, China	*Clarias fuscus*	0.02	1.40	-	27.80	0.37	[54]
	*Channa asiatiea*	0.04	1.02		25.80	0.24	

Cu was detected in all examined fish samples and ranged from 0.306 μg/g to 1.916 μg/g. The highest level of Cu concentration (1.916 μg/g) was found in *Channa striatus*, which was lower than the same fish species of Pahang River, Basin, Malaysia (Table 2) [51]. The lowest concentration of Cu (0.306 μg/g) was found in *Hexanematichthys sagor* species, which is almost the same as the *Puntius bulu* of Chini Lake, Peninsular [52]. Among individual fish species, *Devario regina* exhibited the highest levels of Pb concentration (4.869 μg/g), and it exceeded all the permissible recommended limits. The lowest concentration of Pb was found in *Puntius bulu* (0.378 μg/g), which was even higher than the fish species observed in the Kelantan River, Malaysia (Table 3) [53]. The Pb concentration in *Barbonymus schwanenfeldii* (3.96 μg/g) and *Hexanematichthys sagor* (2.421 μg/g) were also greater than all recommended permissible limits. For *Puntius daruphani* species, the concentration of Pb (1.752 μg/g) exceeded the permissible limit recommended by FAO/WHO 1984 and China National Standard. However, it is still lower than the legislative value of Malaysian Food Regulation 1985 and MAFF 2000. Cd, on the other hand, contributed to the highest trace metal concentration in *Puntius bulu*, which was approximately 0.34 μg/g, and it exceeded all the recommended permissible limits, except for the value according to the Malaysian Food Regulation (1 μg/g). The lowest level of Cd (0.246–0.276 μg/g) in *Hexanematichthys sagor* species was higher compared to fish of Paira River, Bangladesh and Pearl River Delta, China [45, 54]. The concentration of trace metals, such as Fe, was highest in *Devario regina* (140.798 μg/g) species as compared to other fish species, whereas the lowest concentration was found in *Puntius bulu* (32.75 μg/g). All fish species collected from Perak River had Fe concentrations below the permissible limits recommended by FAO/WHO 1984 [48]. Table 3 reveals that the metal contents of fish species in our present study were higher than the other Malaysian studies.

### Level of metals accumulation in the fish from sediment and water

Metals contained in water have a tendency to bioaccumulate in fish tissues, and the accumulation of metals, particularly in muscle tissues of fish could have a direct impact on health throughout the food chain [44]. In this study, average trace metal concentration in fish species from the present study and average trace metal concentration in water from another study which was done at the same time with our present study was used [30]. Bioaccumulation factor of trace metals (Fe, Zn and Pb) in seven fish species in regards to concentration in trace metals are cited in Table 4. The BAF values of trace metals (Fe, Zn and Pb) in all fish species are less than 1000 indicating that there have little or almost no probability of bioaccumulation of such metals in the studied fish species [55]. However, the bioaccumulation of trace metals in fish is carried out via both dietary exposure and waterborne exposure (through gills and skin) [56]. Nevertheless, accumulation of metals from water may vary with the aquatic environment, size and mass of fish, gender, the binding capability of the sampled tissues etc. [57]. *Devario regina* which has the lowest length (12 cm) and mass (6 gm) showed extreme accumulation of metals whereas *Puntius bulu* with the highest length of (35 cm) and weight of (45 gm) exhibited the lowermost accumulation for Zn and Fe as observed by other studies [58]. Furthermore, the higher accumulation of Zn in all fish was observed because it usually remains in the fish muscle as it usually binds with metallothionein (MT) protein and is an essential component of fish muscles cell [59]. Although the accumulation of Pb in fish species is usually caused by food consumption, long-time exposure to Pb might also cause enhanced accumulation in some cases. Since all of the collected fish species are matured enough, it can be assumed that they were exposed to the Pb for a longer period resulting in the enhanced aggregation of Pb in the fish muscle [60, 61].

**Table 4:** BAF values of metals in fish species of Perak River.

Fish species	Fe	Zn	Pb
*Puntius daruphani*	15.69	111.9	175.0
*Barbonymus schwanenfeldii*	11.48	82.0	396.0
*Puntius bulu*	9.52	60.90	380.0
*Hexanematichthys sagor*	9.83	121.43	242.0
*Channa striatus*	42.28	196.40	143.0
*Mystacoleucus marginatus*	26.45	104.46	126.0
*Devario regina*	27.76	280.93	487

BSAF is a parameter describing the accumulation tendency of sediment-associated metals into the tissues of ecological receptors like fish. In this study, BSAF calculation was done from average metal concentrations in fish species from the present study and average metal concentrations in sediments from our previous study, which was done simultaneously (Table 5) [33]. The BSAF values of all metals are less than one (1) meaning that these metals are not accumulated in the fish muscle from sediment directly except with Zn. However, alike BAF *Devario regina* showed maximum BSAF value with an order of Zn> Pb> Cd> Cu > Fe.

**Table 5:** BSAF values of metals in fish species of Perak River.

Fish species	Cd	Cu	Fe x 10^−4^	Zn	Pb
*Puntius daruphani*	0.09	0.05	1.49	0.60	0.06
*Barbonymus schwanenfeldii*	0.10	0.05	1.09	0.44	0.14
*Puntius bulu*	0.11	0.06	0.90	0.32	0.01
*Hexanematichthys sagor*	0.09	0.01	0.93	0.65	0.08
*Channa striatus*	0.09	0.07	4.01	1.05	0.05
*Mystacoleucus marginatus*	0.09	0.04	2.51	0.56	0.04
*Devario regina*	0.09	0.04	2.64	1.50	0.17

### Relationship of trace metals in fish with the feeding habit and environment

Accumulation study from water and sediment to fish species supports the fact that several variables control both bioavailability and accumulation of trace metals in entities while exposed to contamination. Therefore, we also consider the habitat and feedstuffs of these species to find out whether their feeding behaviour is responsible for trace metal accumulation or not. The overall observation is summarised in Table 6. One of the carnivores, *Channa striatus*, tends to move to the flooded area during the rainy season and then migrate to the permanent water like a river. They usually take frogs, snakes, insects, earthworms, tadpoles, small mammals, and crustaceans as diet, most frequently in the rainy season, which might be responsible for the highest quantity of Fe and Cu accumulation in this species. Among the omnivores, *Devario regina* has the highest level of Fe and Zn, which is also found to be associated with their food habits. The higher level of Fe and Zn in *Devario regina*, *Channa striatus* might be originated form the consumption of water bugs and insects because such aquatic insects are sensitive to both biotic and abiotic factors and can accumulate trace metals from sediments and food. A recent study has shown that such bugs can accumulate a higher amount of Fe, Zn and Cu and solubility of Fe from insects which is even higher than beef [62, 63]. Furthermore, from Table 6 it can be elucidated that both *Devario regina* and *Barbonymus schwanenfeldii* contain a higher amount of Pb contents because both species take aquatic plants parts as their food. Recent studies show that aquatic plants can absorb trace metals through the root system from water and sediment and can be ultimately accumulated in the fish species through consumption [64, 65]. Another omnivore, *Puntius bulu*, showed the maximum Cd in the flesh muscle due to uptaking of various aquatic algae, which are usually found to be a great source of Cd by different studies and even some macro-algae are considered as bio-sorbent material for trace metals [66].

**Table 6:** Habitat and common feeds of studied fish species.

Fish species	Habitat	Feeding habit	Common Feed types	Water layer	References
*Puntius bulu*	Freshwater, benthopelagic,	Omnivores	Submerged plants as well as on some filamentous algae and insects, small fish.	Midwater to bottom depths	[67]
*Puntius daruphani*	Freshwater, benthopelagic	Omnivores	Worms and crustaceans	Upland waters	[68]
*Mystacoleucus marginatus*	Freshwater, benthopelagic	Omnivores	Insects, crustaceans, worms, algae and aquatic plants	Bottom depths of rivers	[69]
*Barbonymus schwanenfeldii*	Freshwater, benthopelagic	Omnivores	Aquatic macrophytes and submerged land plants, algae and occasionally insects, small fish, worms, and crustaceans.	Mid-level and bottom of the water	[69]
*Devario regina*	Freshwater, benthopelagic	Omnivores	Ant, beetle, bugs, wasps, plant material like seeds, leaf litter, fish parts etc.	Upper parts of small rivers	[70]
*Hexanematichthys sagor*	Brackish, demersal	Carnivores	Invertebrates and small fish	Upper tidal zone	[71]
*Channa striatus*	Freshwater, brackish, benthopelagic	Carnivores	Frogs, water bugs, insects, earthworms and smaller fish	Not fixed	[72]

However, the various natural trace metals like Pb does not accumulate in fish species. Instead, various anthropogenic point sources are responsible for trace metal accumulation in fish species. The excess accumulation of other elements like Fe, Cd, Zn, and Cu are also caused by various agricultural, domestic, and industrial activities around the Perak river. The major industrial activities around the Perak river are metal processing, metal casting, stainless steel-based factories, welding industries, electroplating industry, electronics industry, shipping industry, battery processing industries, paint industries, textile industries, leather industries, fossil store stations, crushing factories, chemical like ferric chloride plant, wood processing, plastic factories etc. contributing to the discharge of these trace metals which ultimately accumulating into the aquatic species through various routes. Furthermore, numerous newly developed agricultural farm and construction activities and domestic sewage from treatment plants are also contributing to the accumulation of such metals to aquatic organisms.

### Health risk assessment

The fish of the studied region consumed by local people is an important source of protein for them. Therefore, in order to appraise the health risk associated with trace metal contamination of fish inhabiting the Perak River, EDI, THQ, Hazardous Index (HI), and target cancer risk (TR) were estimated.

#### Estimated daily intake

Table 7 reveals that the dietary intake of iron and cadmium exceeded the RfD for seven fish species from the Perak river. Cd is reported as being common in rivers as a result of industrial activities which is detrimental for living organisms [73]. Since EDI of Cd and Fe were at a higher level than the RfD value, the excess consumption of studied fish species in the Perak river should be avoided to prevent health risks to consumers. The intake of Pb in four types of fish species; namely, *Puntius daruphani*, *Barbonymus schwanenfeldii*, *Hexanematichthys sagor* and *Devario regina* exceeded RfD levels as suggested by USEPA, 2000 also [38]. According to these findings, excessive consumption of *Puntius daruphani*, *Barbonymus schwanenfeldii*, *Hexanematichthys sagor*, and *Devario regina* should be avoided to prevent harmful effects caused by Pb accumulation because Pb poisoning is normally ranked as the most common environmental health hazard causing neurotoxicity, nephrotoxicity, and many others adverse health effects [74]. On the other hand, the ingestion of small amounts of contaminated fish that contain Cd over long periods may lead to some form of Cd intoxication and causes pulmonary, renal, skeletal, and reproductive effects as well as cancer [37]. Although Fe is an essential nutrient for the human body, an excessive amount of Fe may lead to breast cancer, colorectal cancer, prostate cancer, lung cancer etc. and eventually to death [75]. Other metals, i.e. Cu and Zn, demonstrated average EDI values that are below RfD level in all fish species. As the average fish consumption by Malaysian people is increasing day by day, it is essential to categorise the fish, which should be avoided from regular uptake.

**Table 7:** Estimated daily intake (EDI), THQ and TTHQ calculated for seven fish species in Perak River.

Fish Species	Estimated daily intake (μg/kg/day)					Target hazard quotients (THQ x10ˉ³)					TTHQ x 10ˉ³
	Cd	Cu	Fe	Zn	Pb	Cd	Cu	Fe	Zn	Pb	
*Puntius daruphani*	0.70	3.57	130.62	83.92	4.37	1.40	0.09	1.86	0.28	1.09	4.73
*Barbonymus schwanenfeldii*	0.72	3.35	95.60	61.50	9.90	1.45	0.08	1.36	0.20	2.47	5.58
*Puntius bulu*	0.85	3.90	79.27	45.67	0.95	1.70	0.10	1.13	0.15	0.24	3.32
*Hexanematichthys sagor*	0.65	0.77	81.87	91.07	6.05	1.30	0.02	1.17	0.30	1.51	4.30
*Channa striatus*	0.67	4.80	352.00	147.3	3.57	1.35	0.12	5.03	0.49	0.89	7.88
*Mystacoleucus marginatus*	0.70	2.95	220.27	78.35	3.15	1.40	0.07	3.15	0.26	0.79	5.67
*Devario regina*	0.70	2.87	231.12	210.7	12.2	1.40	0.07	3.30	0.70	3.04	8.52
RfD (USEPA, 2000) [38]	0.5	40	70	300	4						

#### Target hazard quotients and total target hazard quotients (Noncarcinogenic risk)

All fish species discussed in this study are commonly consumed and used as commercial products by surrounding residents. Therefore, the average trace metal concentrations of fish were used for the calculation of THQ for the surrounding residents. The THQs and TTHQs of the five studied trace metals from consuming seven different fish in the Perak river are listed in Table 7. No THQ value over 1 was observed for the consumption of fish from the Perak river. Iron demonstrated the highest THQ value (5.03 x10^-3^) in *Channa striatus*, whereas Copper displayed the lowest THQ value (0.02x10^-3^) in *Hexanematichthys sagor*. These results indicated that the health risk associated with individual trace metal exposure is insignificant [76]. The TTHQ was also included in this study because humans are often exposed to more than one pollutant and suffer integrated effects. TTHQ was treated as the arithmetic sum of all individual metal THQ values. TTHQ values from all the studied metals were also lower than 1 for all fish, and this result has revealed that the fish species of Perak river are safe for consumption. The findings of this present study are in line with other studies conducted outside Malaysia [25, 42].

However, despite having lower TTHQ value of all fish species, Fe, Pb and Cd are the major contributors of the TTHQ with more than 90% for all fish species. It suggests that local people might experience a certain degree of adverse health effects of the three metals through the studied fish consumption, particularly the *Devario regina* and *Channa striatus*. The other two elements (Cu and Zn) contributed nominally (more or less than 5%) to the TTHQ value of all fish species. However, the TTHQ value of *Devario regina* and *Channa striatus* was higher as compared to the other species because both have higher accumulation tendency of trace metals from water and sediment along with its various diversified food habit and movement tendency.

#### Target cancer risk

The risk of developing cancer from particular trace metals over a lifetime is usually expressed as a target cancer risk. In this study, the target risk (TR) values for lead ranged from 8.03 x 10^−6^ to 1.03 x 10^−4^ (Table 8). Usually, a target risk value below 10^−4^ is considered safe, whereas a value between 10^−3^ and10^-4^ is unacceptable, and a value > 10^−3^ indicates a moderate cancer risk level [77]. Therefore, based on the results of the present study, the potential health risk for the inhabitants from Pb exposure through the consumption of the studied fish is negligible, except for *Devario regina* because the TR value is almost equal to the lower satisfactory limit. The highest TR value was experimented due to the maximum BAF and BCF value in the case of *Devario regina* as described earlier. Notably, TR value of Cd exceeds the satisfactory limit for all fish species, indicating that the studied fish might contribute to high cancer risk in consumers. The tendency of cancer risk for consuming the studied fish was in the following order: *Puntius bulu* > *Barbonymus schwanenfeldii* > *Mystacoleucus marginatus* > *Devario regina* > *Puntius daruphani* > *Channa striatus* > *Hexanematichthys sagor*. Furthermore, it is clear that the cumulative cancer risk value of all fish species was moderate (>10^−3^), and Cd was largely responsible for this result. The cumulative values also suggest that consumption of *Puntius bulu* was the riskiest in this study area, and its consumption would result in approximately 540 cancer cases per 100,000 people exposed [78]. Cd can cause cancer in the kidney by enhancing the transcriptional activity of the metallothionein (MT) coding gene by forming a Cd-MT complex through disrupting calcium metabolism [79]. On the other hand, Pb can cause carcinogenicity through direct DNA damage, inhibition of DNA synthesis and also generate reactive oxygen species leading to oxidative damage to DNA [80]. Therefore, precaution should be taken to control the exposure of Cd in the environment since the levels of the metals were already over the standard limit; otherwise, the ingestion of Cd through the fish species can ameliorate the probability of the cancer growth among the consumers in their lifetime [81].

**Table 8:** Target cancer risk of trace metals due to the consumption of fish from Perak River.

Fish Species	Target cancer risk (L/kg)		Cumulative Target cancer risk (L/kg)
	Pb	Cd	
*Puntius daruphani*	3.72 x 10^−5^	4.43 x 10^−3^	4.46 x 10^−3^
*Barbonymus schwanenfeldii*	8.42 x 10^−5^	4.62 x 10^−3^	4.70 x 10^−3^
*Puntius bulu*	8.03 x 10^−6^	5.35 x 10^−3^	5.36 x 10^−3^
*Hexanematichthys sagor*	5.14 x 10^−5^	4.12 x 10^−3^	4.17 x 10^−3^
*Channa striatus*	3.05 x 10^−5^	4.25 x 10^−3^	4.28 x 10^−3^
*Mystacoleucus marginatus*	2.68 x 10^−5^	4.48 x 10^−3^	4.50 x 10^−3^
*Devario regina*	1.03 x 10^−4^	4.44 x 10^−3^	4.54 x 10^−3^

## Conclusions

The study revealed that the concentration of iron was higher than any other trace metals in fish species of the Perak river. However, the highest EDI was observed in the case of cadmium and lead for *Puntius bulu* and *Devario regina*, respectively. Among all the fish species, the omnivorous species, particularly *Puntius bulu*, exhibited a higher risk of cancer growth to the consumers. Moreover, the results for accumulation investigations revealed that the accumulation of trace metals in fish species might have occurred from the food habit. Considering the importance of food safety in terms of consuming food species of Perak River, and their contamination to the trace metals, this study has provided insights to create awareness among local people about the associated risks.

Moreover, it also suggests that necessary actions should be taken by the authority to prevent the various pollution aspects around Perak river by reducing future contamination levels. However, as this study only considers seven fish species of a particular season, future studies should also consider the other aquatic organisms from different seasons to investigate the effect of the factor on metal accumulation and associated health risks with their consumption. These investigations should be conducted periodically to compare and assess pollution levels throughout the upcoming years.
